# Detection and Quantification of ctDNA for Longitudinal Monitoring of Treatment in Non-Small Cell Lung Cancer Patients Using a Universal Mutant Detection Assay by Denaturing Capillary Electrophoresis

**DOI:** 10.3389/pore.2022.1610308

**Published:** 2022-06-28

**Authors:** Lucie Benesova, Renata Ptackova, Tereza Halkova, Anastasiya Semyakina, Martin Svaton, Ondrej Fiala, Milos Pesek, Marek Minarik

**Affiliations:** ^1^ Center for Applied Genomics of Solid Tumors, Genomac Research Institute, Prague, Czechia; ^2^ Department of Pneumology and Phtiseology, Faculty of Medicine and University Hospital in Pilsen, Charles University, Pilsen, Czechia; ^3^ Laboratory of Cancer Treatment and Tissue Regeneration, Biomedical Center, Faculty of Medicine in Pilsen, Charles University, Pilsen, Czechia; ^4^ Department of Oncology and Radiotherapeutics, Faculty of Medicine and University Hospital in Pilsen, Charles University, Pilsen, Czechia; ^5^ Elphogene, Prague, Czechia; ^6^ Department of Analytical Chemistry, Faculty of Science, Charles University, Prague, Czechia

**Keywords:** liquid biopsy, NSCLC, ctDNA, KRAS mutations, minimal residual disease, capillary electrophoresis, TP53 mutations

## Abstract

**Background:** Observation of anticancer therapy effect by monitoring of minimal residual disease (MRD) is becoming an important tool in management of non-small cell lung cancer (NSCLC). The approach is based on periodic detection and quantification of tumor-specific somatic DNA mutation in circulating tumor DNA (ctDNA) extracted from patient plasma. For such repetitive testing, complex liquid-biopsy techniques relying on ultra-deep NGS sequencing are impractical. There are other, cost-effective, methods for ctDNA analysis, typically based on quantitative PCR or digital PCR, which are applicable for detecting specific individual mutations in hotspots. While such methods are routinely used in NSCLC therapy prediction, however, extension to cover broader spectrum of mutations (e.g., in tumor suppressor genes) is required for universal longitudinal MRD monitoring.

**Methods:** For a set of tissue samples from 81 NSCLC patients we have applied a denaturing capillary electrophoresis (DCE) for initial detection of somatic mutations within 8 predesigned PCR amplicons covering oncogenes and tumor suppressor genes. Mutation-negative samples were then subjected to a large panel NGS sequencing. For each patient mutation found in tissue was then traced over time in ctDNA by DCE.

**Results:** In total we have detected a somatic mutation in tissue of 63 patients. For those we have then prospectively analyzed ctDNA from collected plasma samples over a period of up to 2 years. The dynamics of ctDNA during the initial chemotherapy therapy cycles as well as in the long-term follow-up matched the clinically observed response.

**Conclusion:** Detection and quantification of tumor-specific mutations in ctDNA represents a viable complement to MRD monitoring during therapy of NSCLC patients. The presented approach relying on initial tissue mutation detection by DCE combined with NGS and a subsequent ctDNA mutation testing by DCE only represents a cost-effective approach for its routine implementation.

## Introduction

Lung cancer is the most commonly diagnosed cancer globally, with the highest mortality rate (18.4% of total cancer deaths) [1]. The treatment of lung cancer depends on the histological type, the stage of the disease and the overall physical condition of the patient. Over the past 2 decades the conventional treatment options including surgery, chemotherapy and/or radiotherapy have been supplemented by therapy targeted at tumor-specific aberrations [2]. Furthermore, a notable impact on patient survival has been achieved by introduction of immunotherapy using CTLA-4 or PDL inhibitors [3]. The initial diagnosis relying on histopathology/cytology processing of tumor tissue has been extensively complemented by testing for a presence of molecular markers. The tumor-specific DNA markers including point mutations in *EGFR* or *BRAF* genes, rearrangements involving *ALK* or *ROS1* genes and fusions of *NTRK1*/*2*/*3* genes along with tumor mutation burden (TMB) and PDL-1 RNA expression serve as guides in proper therapy selection. In the event of marker absence patients were so far treated by non-targeted chemotherapy, often using anti-angiogenic agents [2].

In an established routine practice, appropriate selection of targeted treatment by molecular tumor profiling results in a significant improvement of fundamental clinical parameters including extended time to disease progression and overall survival time [4]. The therapy efficacy is further enhanced when the initial proper selection is complemented by close monitoring allowing for timely decisions of interventions in case of emergent therapy resistance. The observation of therapy is primarily relying on imaging techniques including CT or hybrid PET/CT directed at evaluation of tumor morphology (dimensions and volume) and, more recently, also functional parameters including the metabolic activity, tumor vascularization etc. [5].

Use of circulating tumor DNA (ctDNA) has recently demonstrated viability for monitoring of the response to treatment in various solid tumors, including non-small cell lung cancer (NSCLC) [6]. ctDNA is detectable in a form of short DNA fragments released into the bloodstream from the decomposing tumor mass. Due to its exclusive origin in cancerous tissue, ctDNA inherently reflects genetic profile of any present tumor bearing cancer specific DNA mutations [7, 8]. This is used in the methodology of ctDNA monitoring, which is typically directed at detection and quantification of tumor-specific DNA mutations in patient plasma. In a simplified view the ctDNA level (e.g., the number of mutated DNA alleles) reflects the actual volume of tumor mass present in the patient’s body, often referred to as a minimal residual disease (MRD) [9]. MRD monitoring by repeated plasma evaluation in short intervals enables early response by changing or tailoring the treatment for a specific patient. ctDNA monitoring of MRD has been recently applied for anti-EGFR therapy. The typical resulting ctDNA curve showed a decay in ctDNA bearing activating *EGFR* mutation in a sign of initial positive response of the EGFR-sensitive clones followed by an emergence of ctDNA harboring *EGFR* T790M resistant mutation as the EGFR-resistant clone emerges [10–12]. An alternative result has been presented in which a non-targetable mutant (such as *BRAF; KRAS; TP53; STK11*) was tracked resulting in swing-like shape curves tracking the phases of subsequent remissions and progressions of the disease upon administration of multiple lines of systemic therapy [13–15].

The concentration of ctDNA in the circulation is low, typically in the orders of a few ng per mL of plasma [16]. Moreover, its detection is obstructed by an excess of highly similar short DNA fragments coming from non-cancerous body cells, mainly by necrosis due to inflammation or a spontaneous decay by apoptosis [17]. The fraction of ctDNA in a total non-mutated cell-free DNA (cfDNA) could span down to less than 0.05% of mutated (minor) allele fraction (MAF) [18]. Such low ctDNA abundance presents a challenge for detection methodology. Several approaches are currently in use for ctDNA detection. The most universal is indiscriminate comprehensive sequencing of all ctDNA fragments extracted from plasma. This approach, often termed a “liquid biopsy” is exclusively based on improved next-generation sequencing (NGS) technologies ensuring reduction of errors inherent to standard NGS [19]. The upmost advantage of this approach is in its use as surrogate for classic tissue biopsy. In clinical management of lung cancer this is particularly important in situations where specimens acquired by bronchoscopy are not available or do not yield reliable results. While clinically useful, the NGS-based liquid biopsy is very costly with current prices in the order of thousands of EUR per test rendering its use for longitudinal monitoring by repeated testing unfeasible.

Along with the NGS-based liquid biopsy another approach has been presented in which only a DNA mutation found in tumor tissue is subsequently searched for in plasma ctDNA [19]. This “tumor-informed” liquid biopsy is naturally not applicable for initial diagnosis, but with its relatively low cost it is well-suited for repeated testing of MRD. Currently used methods are mainly based on standard mutation detection by quantitative PCR (qPCR) or digital PCR (dPCR). In order to use these for ctDNA detection, the mutant sensitivity is typically enhanced by some means of mutant enrichment, mainly through artificial suppression of amplification of non-mutated (wild type) alleles [20, 21]. Both qPCR and dPCR are performed in an allele-specific format, which means that each such assay is designed to detect only a particular mutation at a particular sequence site. While that is satisfactory for oncogenes, where somatic mutations are mainly localized at specific hotspots, the technique is not applicable for mutant detection in tumor-suppressor genes, whose mutations are typically dispersed across multiple exons [22, 23].

Denaturing capillary electrophoresis (DCE) has been used for routine mutation detection in a variety of solid cancers including colon and rectum [24–26], lung [27, 28], pancreas [29, 30] or brain [31]. The technique is based on heteroduplex formation with subsequent electrophoretic separation to visualize presence of mutant alleles in an abundance of nonmutated wildtype alleles [32]. The method is cost-effective and requires only a very low amount (tens of pg) of input DNA material [25, 31]. In the present work we have applied DCE for prospective monitoring of minimal residual disease in patients with advanced stage of NSCLC. We present a longitudinal observation with frequent sampling during the palliative treatment. We demonstrate clinical utility of the assay for assessment of therapy response as well as early detection of disease progression for use in management of NSCLC patients.

## Materials and Methods

### Patient Inclusion and Clinical Evaluation

A total of 81 patients with histologically confirmed advanced NSCLC (stage III or IV) of adenocarcinoma subtype, treated with standard platinum-based chemotherapy, were eligible for inclusion in this exploratory prospective single center cohort study. Baseline characteristics for 63 patients eligible for ctDNA monitoring (mutation found in tumor tissue) were collected including gender, age, smoking history, ECOG Performance Status, TNM stage, localization of metastases, and chemotherapy regimen (see Table 1). Follow-up data included response to treatment, progression-free survival (PFS) and overall survival (OS) were also collected. Response to treatment was evaluated according to the Response Evaluation Criteria in Solid Tumors (RECIST) 1.1. as complete response (CR), partial response (PR), stable disease (SD) and progressive disease (PD) based on radiological examination [33]. PET/CT or CT scans were performed at the time of diagnosis, after approximately 6 weeks of treatment (after second therapy cycle) and further during the follow-up. PFS and OS were determined as the time elapsed between the initiation of treatment and first documented PD or date of death from any cause, respectively.

**TABLE 1:** Clinicopathological characteristics of NSCLC patients with detected mutation.

Characteristics		Value
Number of patients		63
Age	mean (years)	64.6
	range (years)	40–80
Gender	male	42
	female	21
TNM stage	III	8
	IV	55
Number of metastatic organs	0–1	34
	2–4	29
ECOG Performance status	0	1
	1	53
	2	9
Smoking history	non-smokers	9
	former smokers	16
	smokers	38
First-line chemotherapy	carboplatin + paclitaxel (+ bevacizumab)	32
	cisplatin + pemetrexed	24
	carboplatin + paclitaxel (+ bevacizumab)	6
	cisplatin + vinorelbine	1
RECIST^a^	CR	1
	PR	15
	SD	30
	PD	14
	unknown	3

a The response was evaluated after the 2nd cycle of chemotherapy. CHT, chemotherapy; CR, complete response; PR, partial response; SD, stable disease; PD, progressive disease.

The study complied with the ethical standards of the World Medical Association’s Declaration of Helsinki. The research plan was approved by the Ethical Committee of the Faculty of Medicine in Pilsen, Charles University and University Hospital in Pilsen (Pilsen, Czechia) and informed consent was obtained from all patients.

### Tumor DNA and Plasma-Based ctDNA Extraction and Mutational Analysis

Tumor biopsy material, cytological smear or formalin-fixed paraffin-embedded tissue samples, were processed according to the standard procedures of participating clinical facilities and were obtained from all subjects at study entry. Whole blood samples were collected in stabilization blood collection tubes (Cell-Free DNA BCT, Streck, Inc., United States) before starting systemic therapy (sampling called “P0”—plasma 0, baseline), after approximately 3 weeks of therapy (P1, after first chemotherapy cycle), after second chemotherapy cycle (P2) and then at intervals according to the treatment schedule and/or radiological re-evaluation (P3–P10, follow-up). The plasma fraction was obtained by a two-step centrifugation of whole blood within 6–54 h after collection, and then immediately frozen at −20°C.

Tumor DNA was isolated from all available samples using the GenElute™ Mammalian Genomic DNA Miniprep Kit (Sigma Aldrich, USA) according to the instructions of the manufacturer for the respective tissue material. ctDNA was extracted from 650 µl of plasma collected at each sampling time-point using the QIAamp Circulating Nucleic Acid Kit (Qiagen, Germany) according to the instructions of the manufacturer.

Mutational analysis based on PCR with heteroduplex formation followed by separation by DCE was performed as described previously [31]. Using a tumor-informed approach, only somatic mutations detected in tumor tissue were evaluated in plasma by a personalized MRD assay and the MAF in the ctDNA-positive samples was further calculated. The tissue was initially subjected to mutation analysis using a small 8-amplicon DCE panel—a panel of 8 target regions from most frequently mutated genes in NSCLC including *EGFR*, *KRAS, TP53*, *BRAF* and *PIK3CA*. Analytical sensitivity expressed as the limit of detection (LOD) was evaluated in terms of the minor-allele fraction (MAF) using DNA fragment peak intensities obtained from SeqStudio data using GeneMarker software (Softgenetics, State College, PA, United States) as described previously [31]:

$$MAF=I_{MUTHOMO}+0.5\left(I_{HET1}+I_{HET2}\right)/\left(I_{WTHOMO}+I_{MUTHOMO}+I_{HET1}+I_{HET2}\right)$$

where I_MUTHOMO_ is the signal intensity of the mutant homoduplex peak, I_WTHOMO_ is the signal intensity of the wildtype homoduplex peak, and I_HET1_ and I_HET2_ are the respective signal intensities of the two heteroduplexes. The resulting LODs for each of the tested regions ranging from 0.03% to 0.5% depending on the actual mutation detected.

All details of the DCE panel markers are summarized in Table 2. DNA without any of the initially tested mutations meeting the concentration requirements for NGS testing were then subjected to Illumina MiSeq 67-gene sequencing by ArcherDx VariantPlex Solid Tumor panel (Invitae Corporation, USA). Based on the identified mutation, DCE primers were then designed for amplification of the region around the found mutation (typically 90–200 bp in a total length) followed by a brief optimization of PCR conditions (temperature gradient range 54–68°C) and DCE running temperature (typically within a range from 40 to 60°C). The details of the DCE primer design and running temperature optimization were also described previously [31, 34, 35]. For the DCE the Applied Biosystems SeqStudio Genetic Analyzer instrument (Thermo Fisher Scientific, United States) was used. CE analysis parameters are detailed in Figure 3.

**TABLE 2:** DCE mutation testing panel used in the study.

Marker	Exon number	Target codons	Size of PCR product [bp]	LOD [%]	DCE separation temperature [°C]
*EGFR*	19	746–753	169	0.1	52
*KRAS*	2	12, 13	112	0.03	50
*TP53*	5	170–187	107	0.1	58
	6	187–224	169	0.5	52
	7	225–261	160	0.5	52
	8	262–307	151	0.03	56
*PIK3CA*	9	542	106	0.2	48
*BRAF*	15	600	230	0.05	48

Bp, Base pair; DCE, denaturing capillary electrophoresis; LOD, limit of detection.

## Results

**FIGURE 1:**
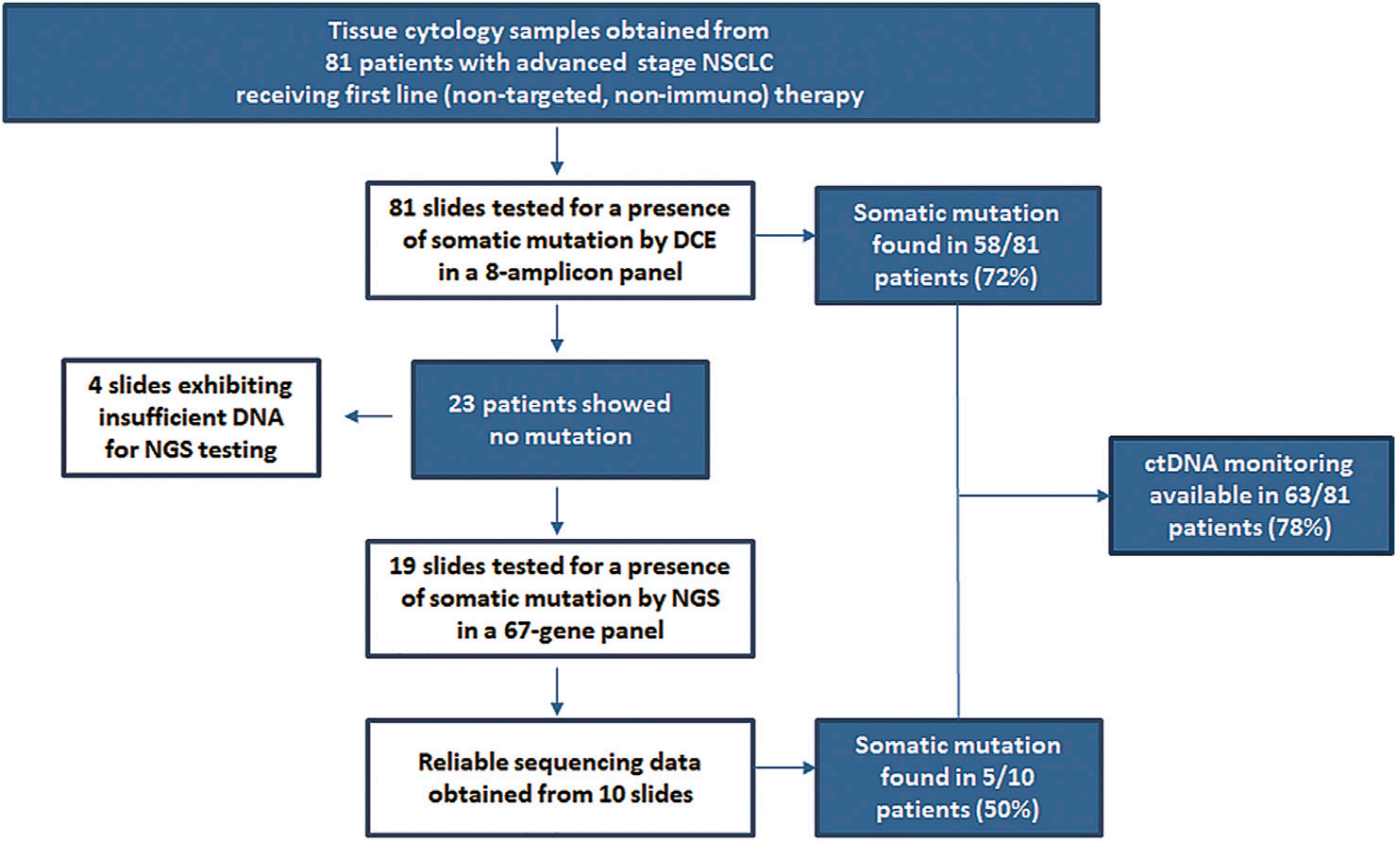
Scheme of multi-tier mutation testing in tissue samples prior to ctDNA monitoring in plasma.

**FIGURE 2:**
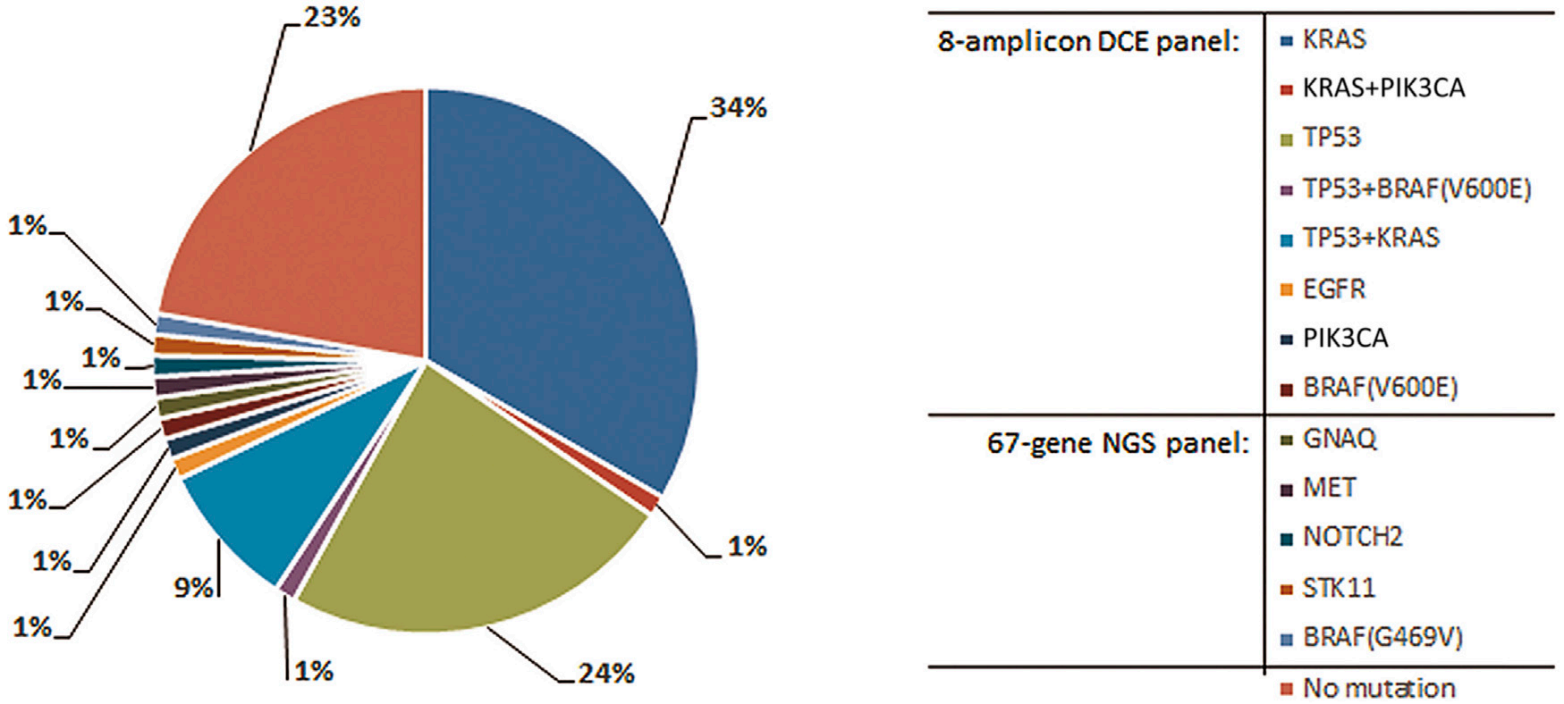
Distribution of mutations found in tissue of 81 NSCLC patients. Specific primers were designed for the mutations identified by NGS to allow for subsequent ctDNA testing in plasma by the DCE method.

**FIGURE 3:**
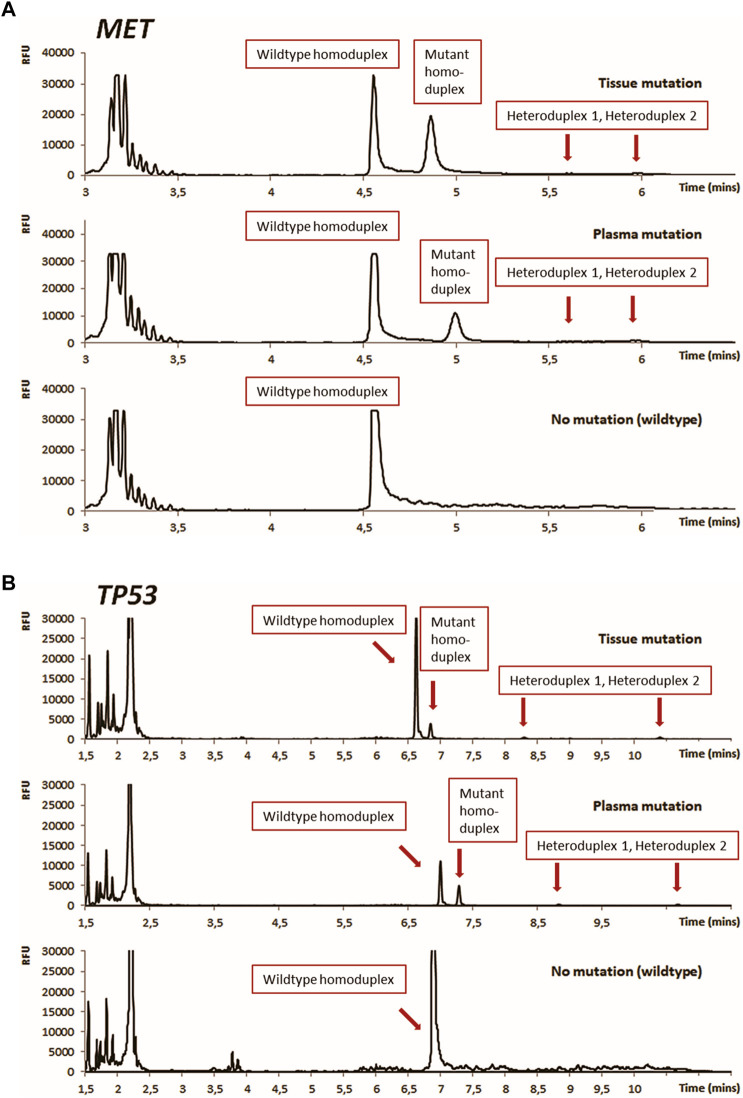
Results of DCE mutation analysis for tissue and plasma illustrated for mutations found in tumor-suppressor genes *MET* **(A)** and *TP53* **(B)**. DCE conditions: Instrument: Applied Biosystems SeqStudio Genetic Analyzer, Injection: 1kV/10 s, Running voltage: 13 kV, Running temperature: 44°C [*MET*, Panel **A**], 54°C [*TP53*, Panel **B**].

Out of a total of 121 patients initially admitted, 81 met the study inclusion criteria (disease stage, histology subtype and therapy setting). The overview of the multi-tier mutation testing is shown in Figure 1. The initial analysis by an 8-amplicon DCE panel revealed somatic mutation in tissue specimens of 58 patients, which represents 72% from the cohort. Of the remaining 23 mutation-negative samples 19 were evaluated as suitable for NGS testing. Using the commercial 67-gene NGS panel additional 5 mutation-positive patients were identified. The spectrum of found mutations shown in Figure 2 corresponds with the expected mutation frequencies with the dominating contribution of mutation in *KRAS* and *TP53* genes (54% in total) often in a combination with other mutations. In 9 patients, 2 mutations were found in the tumor tissue (see Table 3). It should be noted that just upon receiving the complete results from tissue testing, specific DCE primers were designed to allow for monitoring of additional mutations found by NGS. The newly designed DCE assays included *BRAF* Gly469Val, *GNAQ* Tyr101Ter, *MET* Leu971ProfsTer10, *NOTCH2* Trp1529Cys and *STK11* Glu57LysfsTer7 mutations. The DCE result data for selected newly designed *MET* mutation assay is shown in Figure 3A together with a typical example of a mutation detected in the tumor suppressor *TP53* (Figure 3B).

**TABLE 3:** ctDNA quantification in patients having mutations in two different genes in tumor tissue.

Number of patient	Mutations traced in ctDNA	P0	MAF (%)	P1	MAF (%)	P2	MAF (%)
9	KRAS G12V / TP53ex5	O/O	-/-	O/O	-/-	O/O	-/-
14	KRAS G12C / TP53ex7	O/O	-/-	O/O	-/-	-/-	-/-
26	KRAS G12D / TP53ex5	**X**/**X**	21.6/6.9	**X**/O	3.9/-	**X**/O	2.1/-
67	KRAS G12D / TP53ex8	O/O	-/-	O/O	-/-	O/O	-/-
70	KRAS G12C / TP53ex8	**X**/**X**	4.1/<2	**X**/**X**	12.5/<2	**X**/**X**	57.3/29.6
93	KRAS G12C / TP53ex8	**X**/**X**	22.2/11.3	O/O	-/-	-/-	-/-
102	KRAS G12A / PIK3CAex9	**X**/O	15.7/-	**X**/O	6.8/-	**X**/O	7.1/-
105	KRAS G12C / TP53ex5	O/O	-/-	O/O	-/-	O/O	-/-
110	BRAF V600E / TP53ex8	**X**/**X**	40.2/44.9	**X**/**X**	4.1/3.5	**X**/**X**	4.6/2.6

MAF, mutant allele frequency; P0, plasma sample before starting systemic therapy; P1, plasma sample after first chemotherapy cycle; P2, plasma sample after second chemotherapy cycle; X, mutation found; O, no mutation found.

**FIGURE 4:**
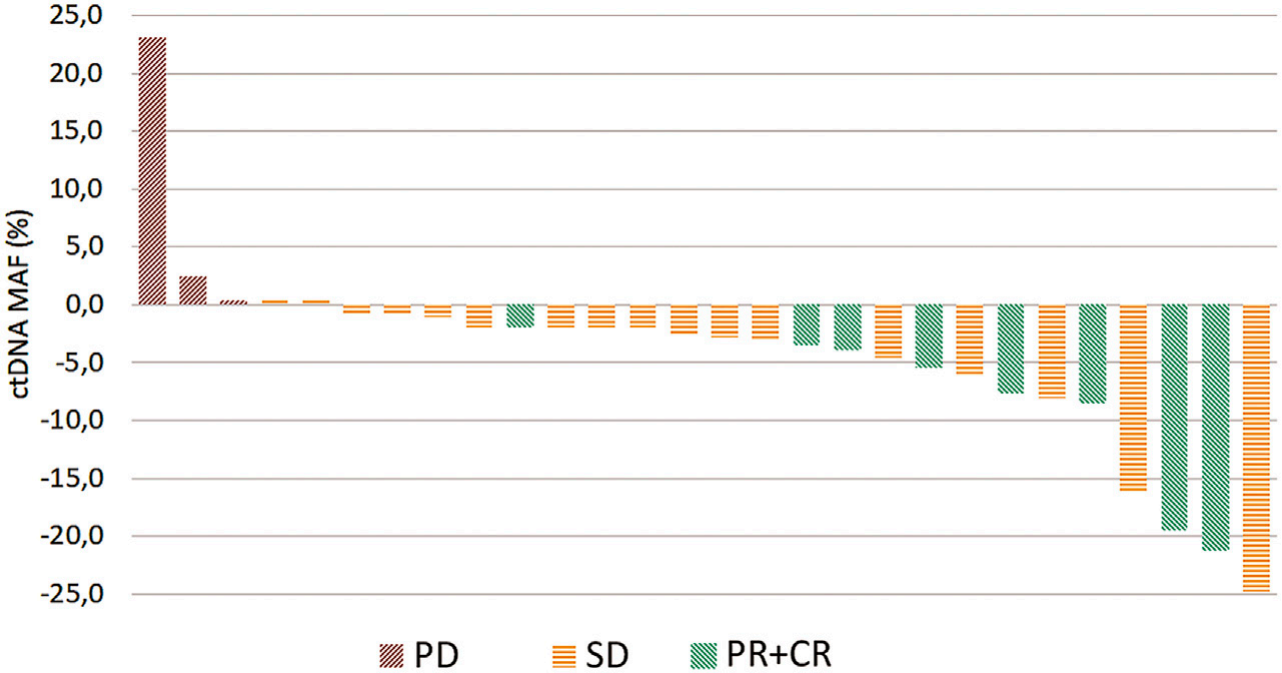
Waterfall plot showing the treatment benefit in 28 patients according to relative change in ctDNA levels between the start of the first and the end of the second cycle of first-line chemotherapy. PD—progression (red), SD—stabilization (yellow), PR—partial + CR—complete response (green).

With the DCE assays optimized for all detected mutations, plasma samples for individual patients were prospectively tested. The baseline P0 samples revealed ctDNA positivity in 36 of the 63 patients. P2 plasma sample was available in 30 of these 36 patients, but MAF could not be determined in 2 patients. During the 6 weeks period upon administration of the first two chemotherapy cycles, the ctDNA levels have been significantly altered in 25 of 28 patients, of whom 23 showed a decrease corresponding to stabilization or remission and 2 an increase corresponding to progression. In 3 patients (1 with progression and 2 with stable disease), ctDNA levels remained virtually unchanged. Overall, changes in ctDNA levels in all patients studied well reflected the RECIST criteria (see Figure 4).

In 9 patients with two mutations found in the tumor tissue, both mutations were monitored and quantified in ctDNA (see Table 3). In individual plasma samples, occurrence of both mutations was very similar. Either always absent (patients 9, 14, 67, 105), or in sample P0 both present and in sample P1 both absent (sample 93), or always present (patients 70 and 110). Moreover, in those last two patients, we uniformly observed an increase (patient number 70) or decrease (patient 110) in the levels of both mutations during P0-P2 samples in response to ongoing chemotherapy. For the remaining 2 patients (26 and 102), a discrepancy in ctDNA detection is likely to occur because the *TP53* and *PIK3CA* mutations were below the LOD.

**FIGURE 5:**
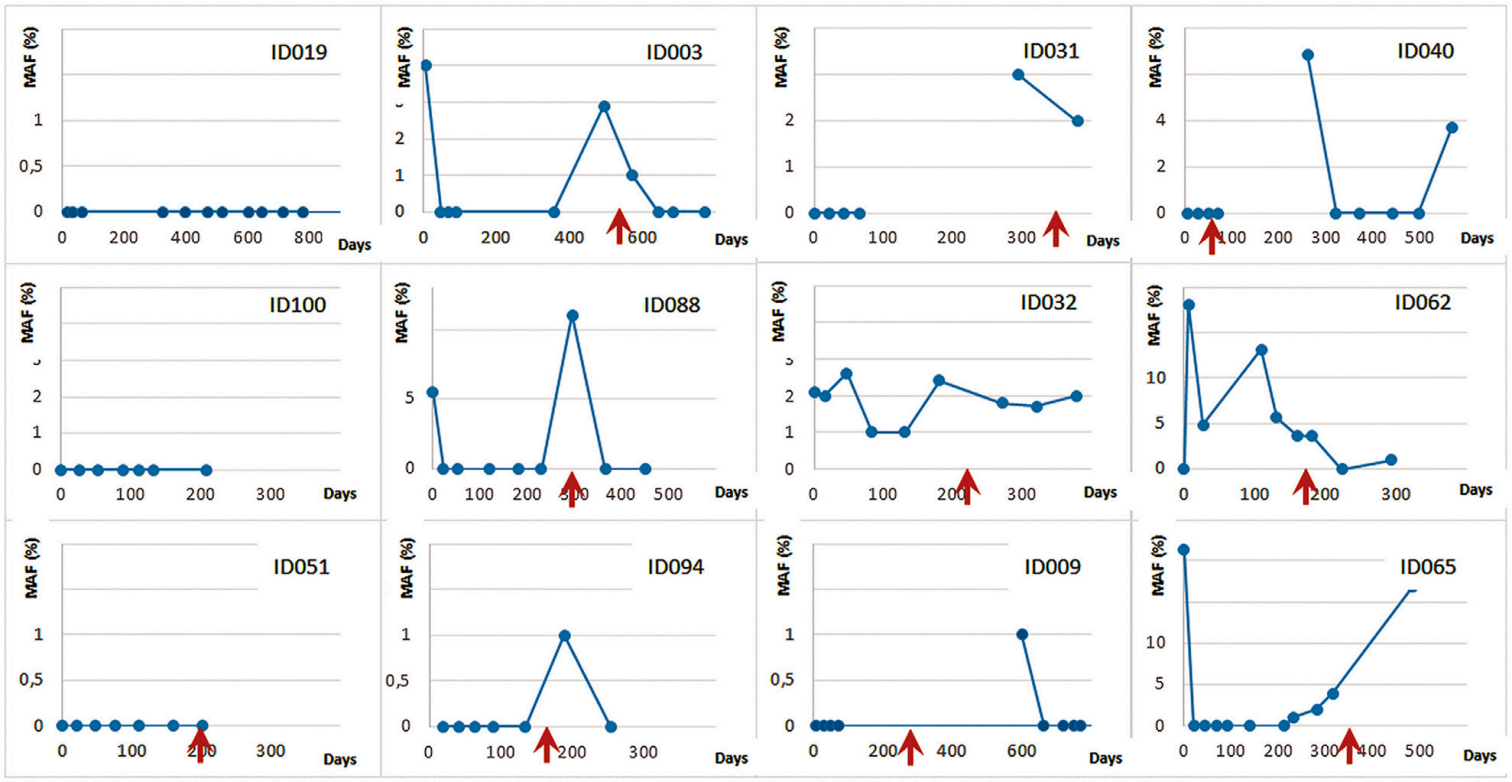
DCE longitudinal MRD monitoring for advanced NSCLC patients undergoing chemotherapy (MAF—% of mutated minor allele fraction). The red arrows denote clinically confirmed disease progression.

For most of the patient in the group the DCE assay was then repeatedly applied in a longitudinal monitoring of MRD as subsequent chemotherapy regimens were administered. In total 340 ctDNA detection analyses were performed among 63 monitored patients over the course of 2 years (follow-up time of individual patients 41–810 days). A clinically confirmed disease progression prompting either a change in regimen or a withdrawal of therapy after an interim partial or complete remission has been detected in 51 patients (mean time to progression 194 days, range 18–803 days). Examples of the long-term follow-up of a subgroup of 12 patients followed for more than half a year and having at least 7 plasma samples are illustrated in Figure 5.

All data acquired during this study is included in Supplementary Table S1.

## Discussion

The central point in the management of patients with advanced NSCLC is the ongoing assessment of the disease situation. This is primarily based on a recurring observation of tumor dynamics by imaging including CT or MRI. This universally accepted approach, however, exhibits certain disadvantages such as an exposure to ionizing radiation in case of CT or a spatial resolution limited to volume differences of several millimeters. A half a decade ago a groundbreaking work on ctDNA testing in NSCLC showed a significant link between changes in tumor volume and plasma ctDNA over time, which opened up prospects for the use of ctDNA in therapy monitoring [36]. The decrease or in some cases complete clearance of tumor specific mutation from ctDNA reflected a positive response to treatment, whereas the rise of ctDNA was directly related to disease progression, with several months lead over radiographic detection and clinical manifestation. In general, such approach is most efficiently applicable if identification of specific therapy-resistant clones is available during an ongoing targeted therapy such as those characterized by *EGFR* mutations T790M or C797S emerging in ctDNA during an ongoing antiEGFR targeted therapy (not applicable in presented patient group).

Over the recent years the most common plasma-based testing in NSCLC has been directed towards evaluation of *EGFR* mutation status as a direct alternative to tissue-based *EGFR* testing. While the ctDNA *EGFR* mutation assays have a stable position in routine diagnostics for prediction of therapy response or detection of therapy resistance, longitudinal monitoring of ctDNA mutations (including the non-targetable ones) has yet to find its full clinical utility. Consequently only a limited number of subsequent studies on longitudinal ctDNA monitoring of tumor-specific mutation in NSCLC has been published [13–15]. In first of them, median of 6 plasma samples from 13 patients with stage IV adenocarcinoma were analyzed during surveillance. Ten patients had *EGFR* sensitising mutations, two *BRAF* V600E mutation, and one patient had a combination of *KRAS*/*TP53*/*STK11* mutations [13]. In the second study, 13 baseline and post treatment ctDNA samples of patients in IV stage of NSCLC were analyzed for the occurrence of hot spot mutations in *EGFR*, *KRAS* and *BRAF* using dPCR [14]. In another study [15], 40 patients with one of the *EGFR*, *KRAS* or *BRAF* mutation was subjected to ctDNA analysis using dPCR at the time of enrollment and at least three follow-up blood samples were collected. The study group included patients in all stages of NSCLC who were treated with different types of treatment (targeted therapy, chemotherapy, immunotherapy, or their combination).

The plasma-based qPCR or dPCR assays typically used in the above mentioned reports were mainly directed at ctDNA detection of oncogenic mutations localized at distinct hotspots such as the relatively small exons 18–21 of *EGFR*, exon 2 of *KRAS* or exon 15 of *BRAF*. For longitudinal MRD monitoring this presents a significant limitation, since a large portion of the somatic DNA point mutations in NSCLC are in tumor suppressors, with absence of such hotspots. According to a recent report somatic mutations in *TP53* tumor suppressor alone account for over 50% of all mutation-positive cases in NSCLC with a strong correlation to tobacco smoke [37].

The DCE method used in this work presents a suitable alternative in targeted monitoring of oncogenic as well as tumor-suppressor mutations. In the current study, ctDNA was monitored in plasma of patients whose tumors were bearing mutations in *TP53*, *APC*, *NOTCH2*, *FGFR1* or *STK11*, among others. All these genes exhibit wide distributions of cancer-associated mutations spread across all coding sequence in a typical mark of a tumor suppressor [38]. Due to its relative simplicity the method can be applied to monitor virtually any ctDNA point mutation after just a very basic PCR amplification optimization. The combination of low noise with wide dynamic range of the SeqStudio detection system enables quantification of CE peak intensities across several orders of magnitude of relative fluorescence units. This allows for readout of the high signal for wildtype fragments next to a low signal for mutant fragments necessary for accurate calculation of MAFs.

In the present study we have evaluated therapy response from the relative change in ctDNA levels from P0 (baseline, prior to therapy) and P2 (after 2nd chemotherapy cycle) sampling. The results, illustrated by a waterfall plot in Figure 4, suggest that such testing may allow for prediction of therapy response already during the first 6 weeks of treatment.

Finally, we have applied the DCE method for repetitive longitudinal testing of patients that have or have not shown ctDNA in baseline sample. The monitoring was performed over the 0.5–2 years therapy period typically covering multiple chemotherapy regimens and in total acquiring from 7 to 11 samples for each patient. The clinical course of the disease was in all cases directly related to the ctDNA dynamics with patients experiencing a lasting remission or stabilization of the disease to those who, during the observation period, showed a disease progression with or without success of the subsequent therapy alterations. A variety of clinical developments could be observed across monitored patients as illustrated in Figure 5. In a subset of patients a continuing positive response to the therapy could be observed as their ctDNA was undetectable during weeks of monitoring (ID019, ID100 and ID051). A more frequently observed course of the disease was characterized by initial response (no ctDNA presence) followed by reappearance of ctDNA several months into the therapy. There a new regimens could either bring a positive effect seen as a reinstated ctDNA elimination (ID003, ID088, ID094, ID009, ID040) or a negative response by continuance of detectable ctDNA (ID032). Occasionally, a lasting progression regardless of the treatment applied could be seen as a slow rise in ctDNA (ID065).

In the current work somatic mutations were first detected in tumor tissue using a two-tier approach in which a small set of frequently mutated oncogenic hotspots was initially evaluated by a smaller DCE panel and the mutation-negative samples were then subjected to a large NGS panel sequencing. For each patient the DCE was then used to detect and quantify the tissue-specific mutation in ctDNA extracted from plasma. A total of 28 patients were subjected to such ctDNA evaluation resulting in a correlation of the ctDNA dynamics with the initial RECIST response approximately 6 weeks into the therapy. A longitudinal MRD monitoring of patients spanning for up to 24 months was also demonstrated. The lower cost of the DCE assay allowed for the ctDNA testing to be performed repeatedly during treatment to monitor the effectiveness of chemotherapy and to detect tumor progression presenting a viable tool useable in routine clinical management on NSCLC. This described approach to MRD monitoring allows for cost-efficient detection of tumor response or, eventual progression before its clinical manifestations and detection by imaging methods.

## Data Availability

The original contributions presented in the study are included in the article/Supplementary Material, further inquiries can be directed to the corresponding author.

## Supplementary Material

Supplementary Table S1 Clinical and experimental data for all patients in the study.
